# Classical back massage is associated with lower heart rate and sustained mean skin conductance during recovery after cognitive load: an exploratory controlled pilot study

**DOI:** 10.3389/fphys.2026.1917630

**Published:** 2026-09-08

**Authors:** Maciej Lachowicz, Iwona Wilk, Igor Hrehorowicz, Szymon Bałamucki, Michał Lisowski, Dariusz Jamro, Grzegorz Żurek, Waldemar Andrzejewski

**Affiliations:** 1 Department of Fundamentals of Physical Therapy and Occupational Therapy, Wroclaw University of Health and Sport Sciences, Wrocław, Poland; 2 Institute of Health Studies, University of Lower Silesia, Wrocław, Poland; 3 Department of Physical Education and Sport, General Tadeusz Kosciuszko Military University of Land Forces, Wrocław, Poland; 4 Department of Biological Principles of Physical Activity, Wroclaw University of Health and Sport Sciences, Wrocław, Poland

**Keywords:** massage therapy, cognitive load, mental workload, electrodermal activity, skin conductance, heart rate, autonomic nervous system, psychophysiology

## Abstract

**Introduction:**

Physiological responses after cognitive load may differ across cardiovascular measures and electrodermal activity (EDA).

**Methods:**

This controlled exploratory pilot study compared heart-rate and phase-averaged mean skin-conductance responses during a 15-min post-task period conducted under two conditions: classical back massage and passive prone rest. After eligibility verification and physiological signal-quality inspection, the final analytical sample included 48 participants: 24 in the classical massage condition and 24 in the passive rest condition. Recordings were segmented into a 5-min baseline, the cognitive task, and three non-overlapping 5-min post-task windows. Heart rate and phase-averaged mean skin conductance were analyzed using mixed-design repeated-measures analyses of variance (ANOVA), with baseline-adjusted values reported descriptively and non-parametric analyses used as supplementary robustness checks.

**Results:**

During the post-task period, the massage group showed a lower heart rate than the passive-rest group across all recovery windows, whereas mean skin conductance remained elevated and was higher during the later windows.

**Discussion:**

These divergent trajectories suggest that cardiovascular and electrodermal measures capture different components of the post-load response and should not be treated as interchangeable indicators of autonomic recovery. The findings provide a basis for confirmatory studies using randomized allocation and active tactile controls, but remain hypothesis-generating because the design did not isolate somatosensory input, interpersonal contact, expectation, posture, or spontaneous recovery.

## Graphical Abstract

**Figure:**
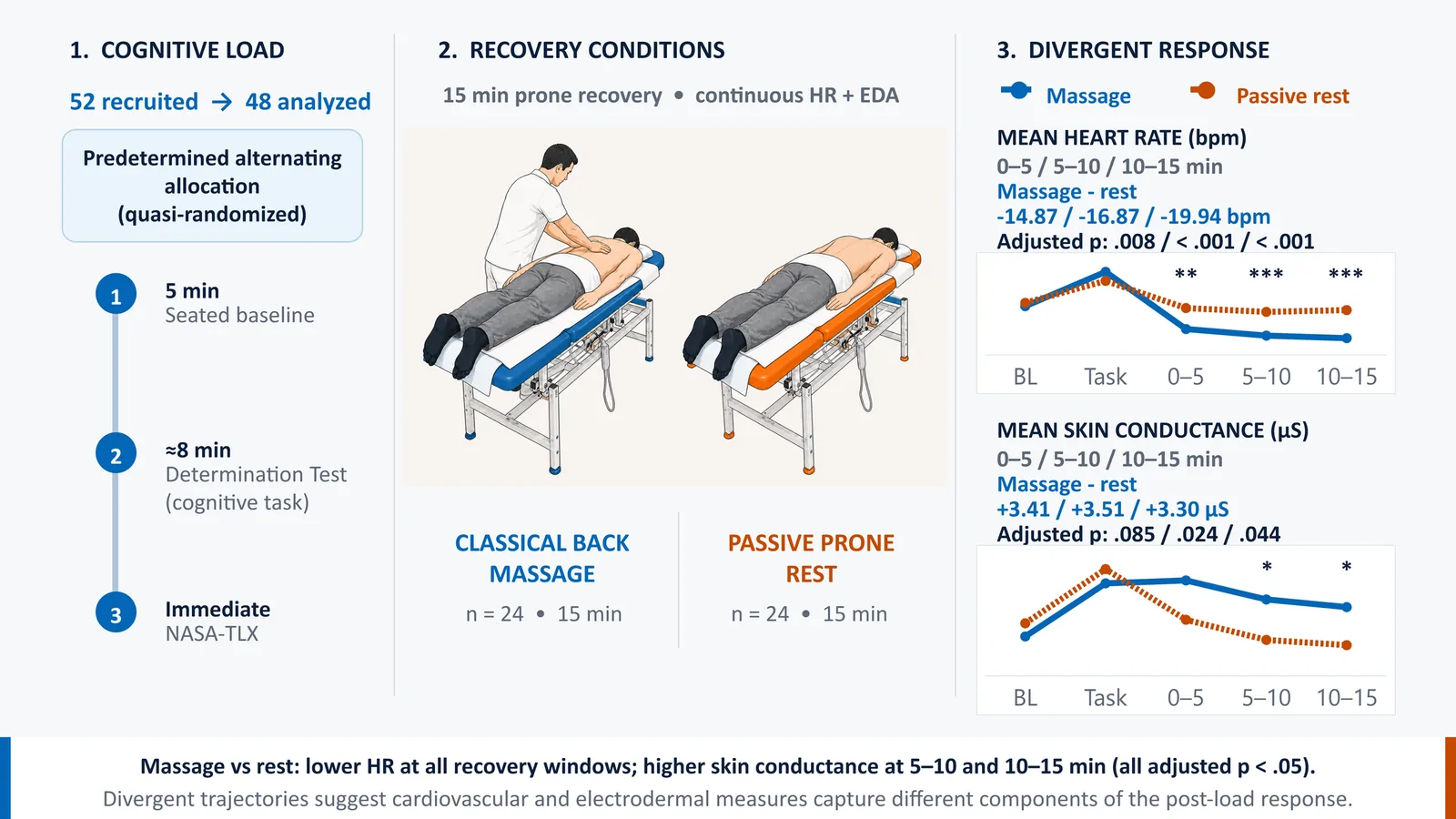

## 1 Introduction

Cognitive performance depends not only on central information processing but also on the capacity to regulate bodily state in response to changing demands, as autonomic indices such as heart rate variability have been linked to executive functioning, decision-making, and broader cognitive regulation (Magnon et al., 2022; Arakaki et al., 2023). Processes such as attention, working memory, inhibitory control, and decision-making are embedded in sensory evidence accumulation, action selection, and behavior-oriented working memory (Fetsch and Noppeney, 2023; van Ede and Nobre, 2023), making cognitive effort and bodily regulation interdependent components of adaptive behavior in cognition–movement contexts (Jansen, 2022; Lustig et al., 2023). Cognitive load is therefore increasingly treated as a multidimensional response involving subjective, behavioral, and physiological components (Longo et al., 2022; Nilsson et al., 2022). Demanding cognitive activity may produce measurable cardiovascular activation and changes in electrodermal activity (EDA) (Al Fawwaz et al., 2024; Suzuki et al., 2024) associated with task demand, fatigue, attentional state, and time-on-task dynamics (Argyle et al., 2021; Csathó et al., 2024). Although most research focuses on activation during task performance, the physiological period following cognitive load remains comparatively underexamined and may follow a different temporal profile (Matuz et al., 2021; Goodman et al., 2025).

Classical massage represents a potentially relevant intervention in this context, as recent massage studies indicate that manual interventions can influence heart rate (HR), blood pressure, and heart rate variability (HRV)-related indices (Fazeli et al., 2020; Kumar et al., 2023). However, massage is not equivalent to quiet rest, as it introduces active sensory input in addition to a resting posture (Kamiya et al., 2021; Köteles et al., 2024; Ruan et al., 2024). This distinction is important when physiological recovery is assessed during, rather than only after, the intervention, particularly because HR and electrodermal activity (EDA), although often used together as indicators of physiological activation, reflect partly distinct regulatory processes. HR indexes cardiac regulation, whereas skin conductance primarily reflects sympathetic sudomotor activity (Amin et al., 2022; Costantini et al., 2023); their combined assessment may therefore clarify whether post-load recovery follows a uniform or marker-specific trajectory (Mazza et al., 2023; Shehu et al., 2023). Within this framework, cognitive load is generally understood as the burden imposed on limited cognitive resources during task performance, particularly when working memory, attention, inhibition, and executive control are required (Sweller, 1988; Sweller et al., 1998; Paas et al., 2003). In cognitive load theory, excessive demands may interfere with efficient information processing, especially when task complexity, time pressure, or irrelevant processing requirements exceed available capacity (Sweller et al., 2011; Young et al., 2015). Cognitive overload can therefore be conceptualized as a state in which cognitive demands surpass regulatory resources, resulting in increased effort, reduced efficiency, performance instability, or physiological activation (Hockey, 1997; Young et al., 2015).

The state of the art in cognitive load assessment is multidimensional. Subjective measures, such as the NASA Task Load Index (NASA-TLX) and mental effort ratings, remain widely used as they capture perceived task demands and individual appraisal (Hart and Staveland, 1988; Paas et al., 2003). However, subjective ratings are retrospective and may be influenced by motivation, self-awareness, fatigue, or response bias (Paas et al., 2003; Ayres et al., 2021). Behavioral measures, such as accuracy, reaction time, and error rate, provide objective information about task performance, but they may underestimate cognitive strain when participants preserve performance through compensatory effort (Hockey, 1997; Young et al., 2015). Physiological measures are therefore increasingly used alongside subjective and behavioral indicators, especially in digitally mediated cognitive–motor contexts such as electronic sports (esports) and virtual reality (VR) -based training, where performance relies on attention, visuospatial working memory, reaction speed, and psychomotor coordination (Lachowicz et al., 2024; Lachowicz et al., 2025).

Physiological workload research has focused on cardiovascular measures, EDA, respiration, eye tracking, electroencephalography (EEG), and multimodal sensor combinations (Charles and Nixon, 2019; Tao et al., 2019; Kosch et al., 2023). Reviews show that many physiological markers are sensitive to changes in workload, but no single measure can be considered a universal index of cognitive load across tasks, populations, and contexts (Charles and Nixon, 2019; Tao et al., 2019). This limitation is particularly relevant for heart rate and skin conductance. Heart rate may reflect cognitive effort, arousal, posture, respiration, emotional activation, or recovery dynamics, whereas EDA primarily reflects sympathetic sudomotor activity and may be influenced by cognitive effort, affective arousal, novelty, and sensory stimulation (Critchley, 2002; Boucsein, 2012; Laborde et al., 2017; Bari et al., 2023). Therefore, these markers are informative but not interchangeable.

Most cognitive-load studies focus on physiological activation during task performance, whereas the post-task period is less frequently examined (Meijman and Mulder, 1998; Hockey, 2013). This is relevant given that real-world cognitive functioning involves repeated cycles of demand and recovery, and post-task regulation may influence subsequent readiness, fatigue resistance, and cognitive–motor performance (Meijman and Mulder, 1998; Sonnentag and Fritz, 2015). Investigated strategies range from reducing unnecessary task demands through improved information and task design (Sweller et al., 1998; Castro-Alonso et al., 2021) to rest breaks, breathing exercises, mindfulness-based interventions, HRV biofeedback, physical activity, sleep hygiene, and structured recovery routines (Lehrer et al., 2020; Cao et al., 2022). Their effects and practical requirements vary: micro-breaks appear more consistently beneficial for fatigue and vigor than for performance (Albulescu et al., 2022), whereas mindfulness-based interventions and HRV biofeedback may require training, adherence, equipment, or continued cognitive engagement (Cao et al., 2022). This heterogeneity provides a rationale for examining accessible, non-pharmacological strategies that can be applied immediately after demanding cognitive activity.

Massage is commonly used to support relaxation, reduce perceived stress, and facilitate recovery. Unlike quiet rest, it combines tactile, pressure-related, proprioceptive, and broader somatosensory input (Field, 2014; Field, 2016), with proposed mechanisms involving mechanoreceptor activation, affective-touch pathways, pain modulation, vagal processes, and perceived bodily safety or comfort (Diego and Field, 2009; Field et al., 2010; Nelson, 2015). Meta-analytic evidence suggests that single sessions may reduce state anxiety, blood pressure, and heart rate, although effects on cortisol are inconsistent (Moyer et al., 2004) and generally small (Moyer et al., 2011). Considerable heterogeneity in populations, protocols, pressure, duration, comparator conditions, and outcome measures nevertheless limits strong mechanistic conclusions (Nelson, 2015; Field, 2016). Physiological responses may also differ according to stimulus characteristics: moderate-pressure massage has been associated with parasympathetic-related and cardiovascular changes, whereas light touch or sensory stimulation may produce different responses (Diego and Field, 2009; Field et al., 2010). Consequently, lower HR alongside sustained mean skin conductance during ongoing massage is theoretically plausible rather than inherently contradictory (Critchley, 2002; Boucsein, 2012).

Although massage has been studied in relation to anxiety, pain, stress, blood pressure, and general relaxation, its role in recovery after experimentally induced cognitive load remains insufficiently examined. Existing cognitive-load studies often focus on task-related activation, whereas massage studies often focus on clinical symptoms, perceived stress, pain, or general autonomic outcomes. Few studies have compared cardiovascular and EDA during post-task massage and passive rest while analyzing both markers simultaneously. The present study addresses this gap by examining heart rate and mean skin conductance across baseline, cognitive-task, and post-task phases under two different conditions.

Accordingly, the present study compared post-task HR and mean skin-conductance responses between classical back massage and passive prone rest after cognitive load in healthy young adults. We expected the cognitive task to increase physiological activation relative to baseline and examined whether HR and mean skin-conductance trajectories during the post-task period differed between the two conditions. Given the exploratory design, the study aimed to generate preliminary, hypothesis-forming evidence regarding condition-associated physiological patterns after cognitive load.

## 2 Materials and methods

### 2.1 Participants

An *a priori* sample size calculation was conducted in G*Power for a repeated-measures analysis of variance (ANOVA) with a within–between interaction. The original planned analytical structure included two groups and three repeated measurements. The calculation assumed a medium effect size (f = 0.25), α = .05, statistical power of.95, an assumed correlation among repeated measures of.50, and a nonsphericity correction of ϵ = 1. Under these assumptions, the required total analytical sample size was estimated at N = 44. To account for possible exclusions related to eligibility criteria, participant withdrawal, or incomplete repeated-measures physiological data, an anticipated dropout/data-loss rate of approximately 15% was included; therefore, the planned recruitment target was set at N = 52. The final physiological analytical sample of N = 48 exceeded the originally planned minimum analytical sample size. The later subdivision of the recovery period into three non-overlapping 5-min windows was treated as an analytical refinement consistent with the exploratory character of the study.

**Figure 1:**
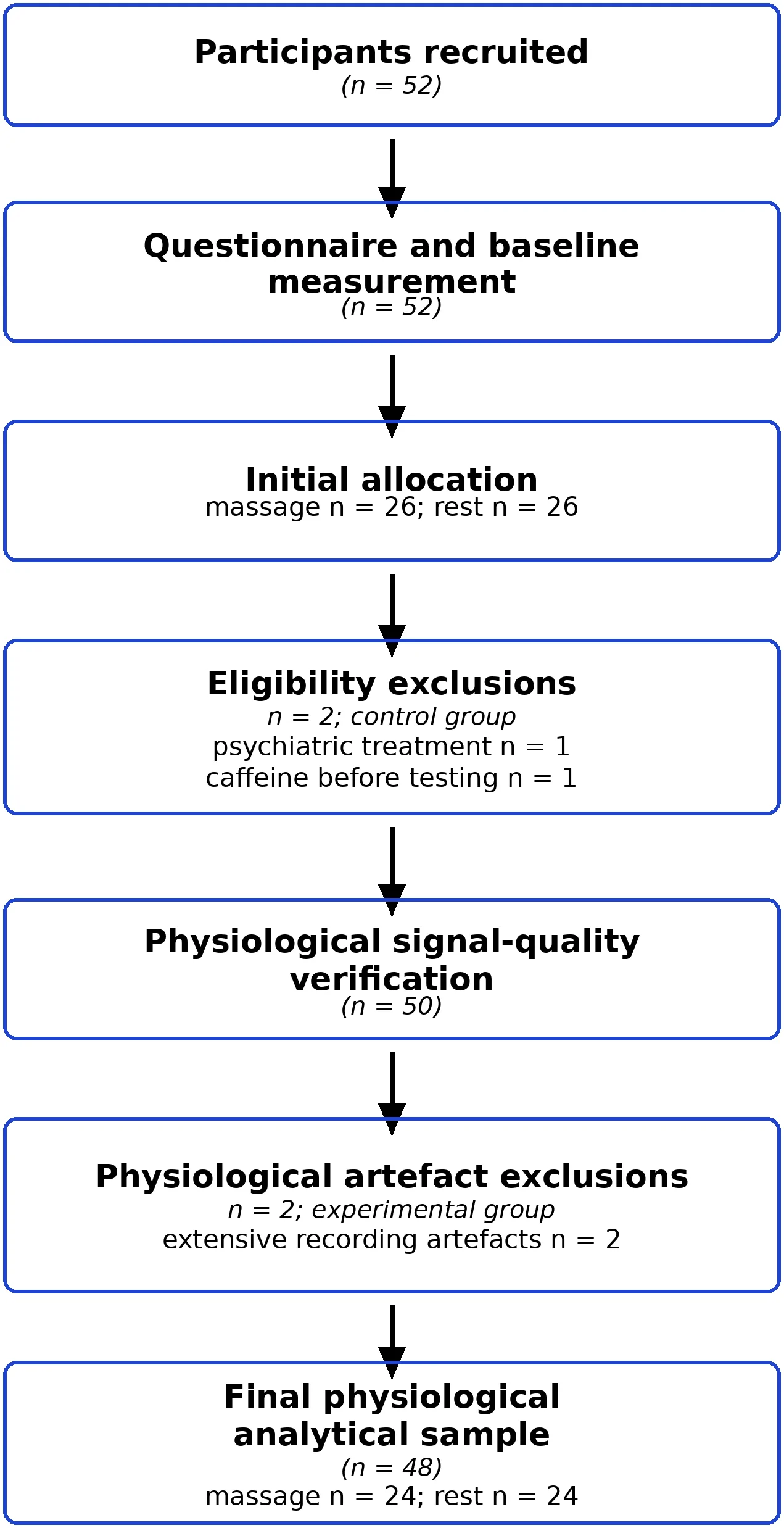
Participant flow diagram showing recruitment, allocation, eligibility-based exclusions, physiological signal-quality exclusions, and the final physiological analytical sample.

Participants were allocated sequentially to the massage or passive-rest condition using a predetermined alternating sequence, constituting quasi-random rather than true random allocation. This pragmatic procedure was selected during the exploratory pilot phase to maintain approximately balanced condition sizes throughout sequential recruitment. The sequence was established before data collection and was applied without modification based on participant characteristics. A total of 52 participants were recruited and allocated sequentially to the experimental (n = 26) or control (n = 26) condition. Two control participants were excluded following eligibility verification (psychiatric treatment, n = 1; caffeine consumption before testing, n = 1), and two experimental-group recordings were excluded because of extensive physiological artefacts. **Figure 1** presents the complete participant flow, including recruitment, allocation, eligibility-based exclusions, physiological signal-quality exclusions, and inclusion in the final analytical sample. The final analytical sample comprised 48 participants: 24 in the experimental group (13 women and 11 men) and 24 in the control group (15 women and 9 men), with a mean age of approximately 19.5 years. No statistically significant between-group differences were observed in baseline characteristics (**Table 1**).

**Table 1:** Baseline demographic, sleep, fatigue, and self-reported state characteristics of the final sample.

Variable	Experimental group	Control group	p
Sex, women/men	13/11	15/9	.558
Age, years	19.42 ± 0.90	19.63 ± 1.35	.811
Body height, cm	176.73 ± 10.95	171.63 ± 9.60	.083
Body mass, kg	70.20 ± 15.15	64.73 ± 12.81	.258
BMI, kg/m²	22.11 ± 2.76	21.80 ± 2.54	.503
Sleep duration (h)	6.27 ± 1.56	6.58 ± 1.56	.610
Sleep quality (0-10)	6.31 ± 2.07	6.46 ± 2.26	.821
Physical fatigue (0-10)	4.27 ± 2.27	4.13 ± 2.13	.730
Sleepiness (1-9)	4.77 ± 1.56	4.30 ± 1.74	.318
Mental fatigue (0-10)	4.77 ± 2.03	4.39 ± 1.75	.435
Stress (0-10)	3.65 ± 2.73	3.50 ± 2.50	.945
Pain/discomfort (0-10)	1.46 ± 1.53	1.42 ± 1.91	.627

Values are presented as mean ± standard deviation, except for sex, which is presented as counts. Between-group comparisons for continuous variables were performed using Mann–Whitney U tests. Sex distribution was compared using the chi-square test. BMI, body mass index.

Participants were screened before the experimental procedure using a structured health and eligibility questionnaire. The screening covered general health status, neurological and psychiatric history, other diagnosed diseases that could affect participation or physiological responses, medication or substance use affecting the central or autonomic nervous system, contraindications to massage, caffeine and nicotine intake in the 4 h preceding the study, and intensive physical exercise in the 12 h preceding the study. Participants were also asked about their willingness to participate and provide informed consent.

The inclusion criteria were: age of at least 18 years, self-reported good general health, absence of contraindications to participation in the experimental procedure and classical massage, and provision of written informed consent. The exclusion criteria were: neurological, psychiatric, or other diseases that could affect the course of the study or physiological responses; use of medication or substances influencing the autonomic nervous system; contraindications to massage, including skin lesions or inflammatory conditions; lack of consent or withdrawal from the study; caffeine or nicotine consumption within 4 h before testing; and intensive physical exercise within 12 h before testing.

Eligibility was verified on the basis of participants’ self-reported questionnaire responses. After completion of data collection, the screening questionnaires were reviewed again before results synthesis or statistical analysis. All participants provided informed consent before participation. The study adhered to the ethical standards outlined in the Helsinki Declaration of 1964 and its subsequent revisions. The study was approved by the Ethics Committee of Wroclaw University of Health and Sport Sciences; approval number: 46/2025. Full ethical approval documentation is available in **Supplementary File 1**.

### 2.2 Measurements

The measurements included baseline self-reported state variables, subjective workload, task-performance indices, cardiovascular and electrodermal measures. This approach was used to characterize participants’ pre-task psychophysiological state, perceived task demand, cognitive-task performance, and cardiovascular responses and changes in EDA across the experimental phases. Before the experimental procedure, participants completed a structured baseline questionnaire. The questionnaire included demographic and anthropometric information, including age, sex, body height, body mass, body mass index (BMI), and handedness. Participants also reported sleep duration during the previous night, perceived sleep quality, current physical fatigue, sleepiness, mental fatigue, stress, and pain/discomfort. Sleep quality, physical fatigue, mental fatigue, stress, and pain/discomfort were assessed using numerical 0–10 scales. Higher scores indicated greater intensity of the assessed state, except for sleep quality, where higher values indicated better perceived sleep quality. Sleepiness was assessed using the 1–9 Karolinska Sleepiness Scale, with higher scores indicating greater sleepiness. These variables were used to characterize baseline status and to compare the experimental and control groups before the intervention.

Subjective workload was assessed using the NASA Task Load Index (NASA-TLX) (Hart and Staveland, 1988). The NASA-TLX includes six dimensions: mental demand, physical demand, temporal demand, performance, effort, and frustration. Each dimension was scored on a 0–100 scale. In the performance dimension, lower values indicated better perceived performance, with 0 representing perfect performance. The NASA-TLX was used to characterize the subjective workload induced by the cognitive task and to complement physiological responses.

Task performance during the cognitive-load induction procedure was characterized using Determination Test (DT) performance indices. The analyzed DT variables included median reaction time, number of correct responses, number of incorrect responses, number of omitted responses, and accuracy. These indices were used to verify whether the experimental and control groups differed in task execution during the cognitive-load phase.

Physiological signals were recorded continuously using a Consensys/Shimmer3 GSR system with ConsensysPRO v2.0.23 software. EDA and raw photoplethysmography (PPG) were acquired at 128 Hz. The recorded channels comprised skin conductance, raw PPG, PPG-derived heart rate (HR), PPG-derived inter-beat intervals, accelerometer, and gyroscope data. HR was derived automatically from the raw PPG signal using the built-in Consensys PPG-to-HR algorithm. No additional offline filtering, smoothing, or resampling was applied to the EDA signal. The primary physiological outcomes were phase-averaged mean skin conductance and HR. Skin conductance was expressed in microsiemens (µS), whereas HR was expressed in beats per minute (bpm). Mean skin conductance was calculated as the arithmetic mean of retained valid samples within each predefined experimental phase and recovery window. Formal tonic–phasic decomposition of EDA was not performed; consequently, the reported outcome does not distinguish between tonic skin conductance level and phasic skin conductance responses. Phase-level HR values were similarly calculated as the arithmetic mean of retained valid PPG-derived HR samples within each analytical phase and recovery window.

PPG-derived inter-beat intervals were recorded automatically by the acquisition system. However, pulse rate variability (PRV) was not a prespecified outcome, and the exported interval series were not processed using the dedicated beat-level validation and artefact-correction procedures required for reliable variability analysis, particularly during ongoing manual stimulation. Signal quality sufficient for calculating phase-averaged HR does not necessarily establish the reliability of interval-to-interval variability. Therefore, *post hoc* PRV analyses were not performed. Skin-conductance electrodes were placed on the proximal phalanges of the index and middle fingers of the non-dominant hand. The optical pulse sensor was placed on the left earlobe. Accelerometer and gyroscope channels were retained to support the identification of movement-related signal disturbances but were not treated as primary outcomes. Representative synchronized recordings illustrating signal quality during massage and movement-related disturbance during the task-to-recovery transition are presented in **Supplementary Figure 1**.

After acquisition, physiological recordings were segmented offline using timestamped procedural annotations recorded during each experimental session. For each participant, timestamps identified the start of the resting baseline, the start and end of the cognitive task, the start of the assigned recovery condition, and the end of the recovery period. These timestamps were matched to the corresponding time points in the exported physiological recording, and phase boundaries were assigned to the nearest available sample. Transition periods related to instruction, repositioning, sensor adjustment, or preparation for recovery were excluded *a priori* from all analytical phases.

EDA samples were considered invalid when the acquisition system returned a missing or unavailable conductance value, when the recorded value fell outside the measurable range of the Shimmer3 GSR sensor (0.2–125 µS), or when a discontinuity in skin conductance occurred concurrently with a movement-related disturbance in the synchronized accelerometer or gyroscope channels. Changes in the inertial channels without a corresponding disruption of EDA were not sufficient for exclusion. No amplitude-based exclusion threshold was applied to skin-conductance values within the measurable range. For HR, negative placeholder values generated by the acquisition software and values exceeding 200 bpm were treated as invalid and excluded from phase-level averaging. No interpolation was applied to excluded or unavailable samples. A recording was excluded at the participant level when signal loss or artefacts prevented calculation of a phase mean for at least one primary physiological outcome in any required analytical phase. Two experimental-group recordings met this criterion and were excluded from the physiological analysis.

Baseline-adjusted delta values were calculated as phase value minus baseline value. Task-related change was defined as the difference between the cognitive task phase and baseline, whereas recovery-related change was defined as the difference between each recovery window and baseline. These delta values were used descriptively to characterize the magnitude and direction of task-related activation and post-task recovery. All measurements were conducted in the same room and within the same daytime window, between 10:00 AM and 3:00 PM. Experimental and control procedures were conducted under comparable room conditions.

### 2.3 Experimental procedure

Each participant completed the experimental session individually under controlled laboratory conditions. Before the experimental recording, participants completed an initial questionnaire used for sample characterization and questionnaire-based analytical eligibility verification. In accordance with the approved study procedure, all consenting participants were allowed to complete the full experimental session. Questionnaire-based exclusion criteria were applied after completion of the procedure and before statistical analysis, whereas physiological signal-quality exclusions were applied after inspection of the recorded data. This approach ensured that the final analytical sample met the predefined questionnaire- and signal-quality-related eligibility criteria.

**Figure 2:**
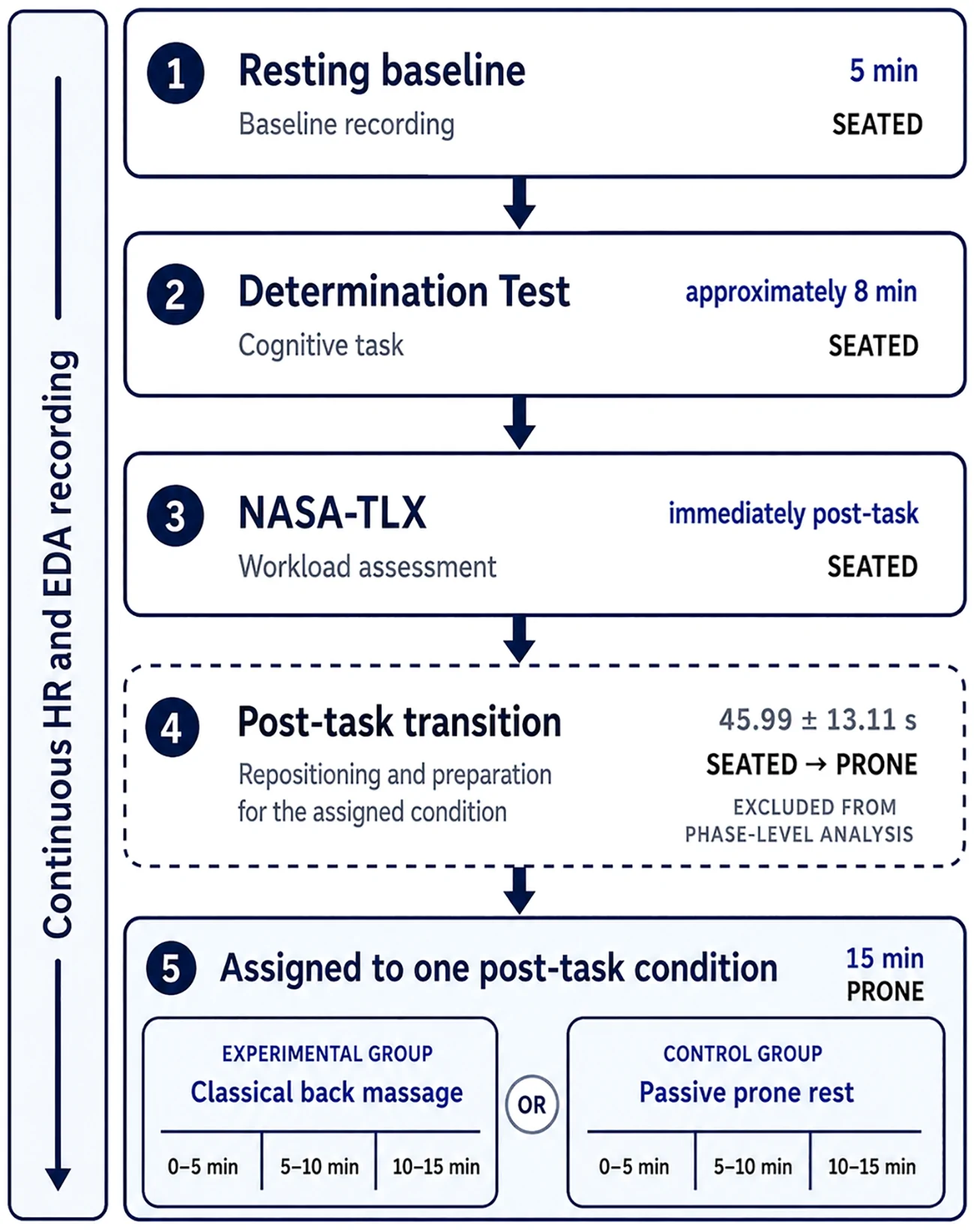
Schematic overview of the experimental protocol, including the sequence and duration of each study phase.

After the initial questionnaire, physiological sensors were attached as described in the physiological recording section. Signal quality was checked before recording, and participants remained seated quietly during a short stabilization period. As shown in **Figure 2**, the analytical recording began with a 5-min seated baseline, followed by standardized Determination Test instructions and the adaptive long form of the Determination Test, which lasted approximately 8 min. Immediately after completing the cognitive task, participants completed the NASA-TLX to assess perceived workload associated with the Determination Test. After completion of the NASA-TLX, participants were repositioned from the seated task position to the prone recovery position. This transition interval comprised repositioning and preparation for the assigned recovery condition and ended when either classical massage or passive prone rest began. In the final physiological analytical sample, transition duration was available for all 48 participants and averaged 45.99 ± 13.11 s. Its mean duration was 44.97 ± 13.46 s in the control group and 47.00 ± 12.95 s in the experimental group, with no statistically significant between-group difference (U = 243.0, p = .359). The transition interval was excluded from phase-level analysis.

The recovery phase began when the participant had assumed the prone position and the assigned recovery condition started. Participants completed 15 min of either classical back massage in the experimental group or passive prone rest in the control group. For phase-level analysis, the recovery period was divided into three consecutive, non-overlapping windows: 0–5, 5–10, and 10–15 min. After recovery, the sensors were removed and the recordings were exported for preprocessing.

### 2.4 Cognitive task

The cognitive task was used as a standardized procedure to induce a controlled period of high cognitive load before the recovery condition. Cognitive load was induced using the Determination Test (DT) from the Vienna Test System (SCHUHFRIED GmbH, Mödling, Austria), test version S2: adaptive long form.

The DT is a computer-based reaction and stress-tolerance task in which participants respond to rapidly changing visual and acoustic stimuli according to predefined stimulus–response mappings. During the task, stimuli are presented in continuous succession on the screen and through auditory channels. Visual stimuli were displayed on a 17.3-inch liquid-crystal display (LCD) screen with a resolution of 1920 × 1200 pixels, positioned approximately 45 cm in front of the seated participant. Auditory stimuli were delivered through speakers. Depending on the stimulus type, the participant is required to select the corresponding response as quickly and accurately as possible using the response interface. In standard DT administration, this may include responding to color stimuli, visual signals, and sounds by pressing the appropriate buttons or keys assigned to each stimulus category.

The task requires continuous monitoring of incoming information, rapid stimulus discrimination, maintenance of response rules, and immediate response selection under time pressure. In the adaptive long form, task difficulty is adjusted dynamically to the participant’s performance level, which supports sustained cognitive demand throughout the procedure. The DT was therefore selected for the present protocol because its structure combines sustained attention, working memory for response mappings, response selection, inhibitory control, decision-making, and psychomotor speed, making it suitable for inducing a cognitively demanding state before the subsequent recovery phase.

### 2.5 Intervention

After completion of the cognitive task, participants entered the recovery phase according to their assigned group. Participants in the experimental group received a single 15-min relaxation-oriented classical back massage, whereas participants in the control group underwent a 15-min passive rest condition without manual stimulation.

The massage intervention was delivered by one of three trained providers. Each provider had completed the same six-month classical massage course within the physiotherapy curriculum and had a comparable level of practical experience. The use of three providers was therefore standardized by common training background and by application of an identical predefined massage protocol across participants. Before the recovery phase, the massage table was prepared in the same way for all participants and covered with a clean sheet. Participants were positioned prone immediately after the post-task transition, and the intervention began only after the participant had assumed a stable and comfortable position.

To standardize skin contact and manual friction during the intervention, the same massage lubricant was used in all experimental sessions. A commercially available vitamin massage oil was applied in each case (Ziaja, vitamin massage oil, 500 ml). The lubricant was used only within the back-massage area and was not applied to the hands, fingers, earlobe, or any physiological sensor-placement sites. No other massage oils, creams, warming preparations, or topical agents were used during the intervention.

**Figure 3:**
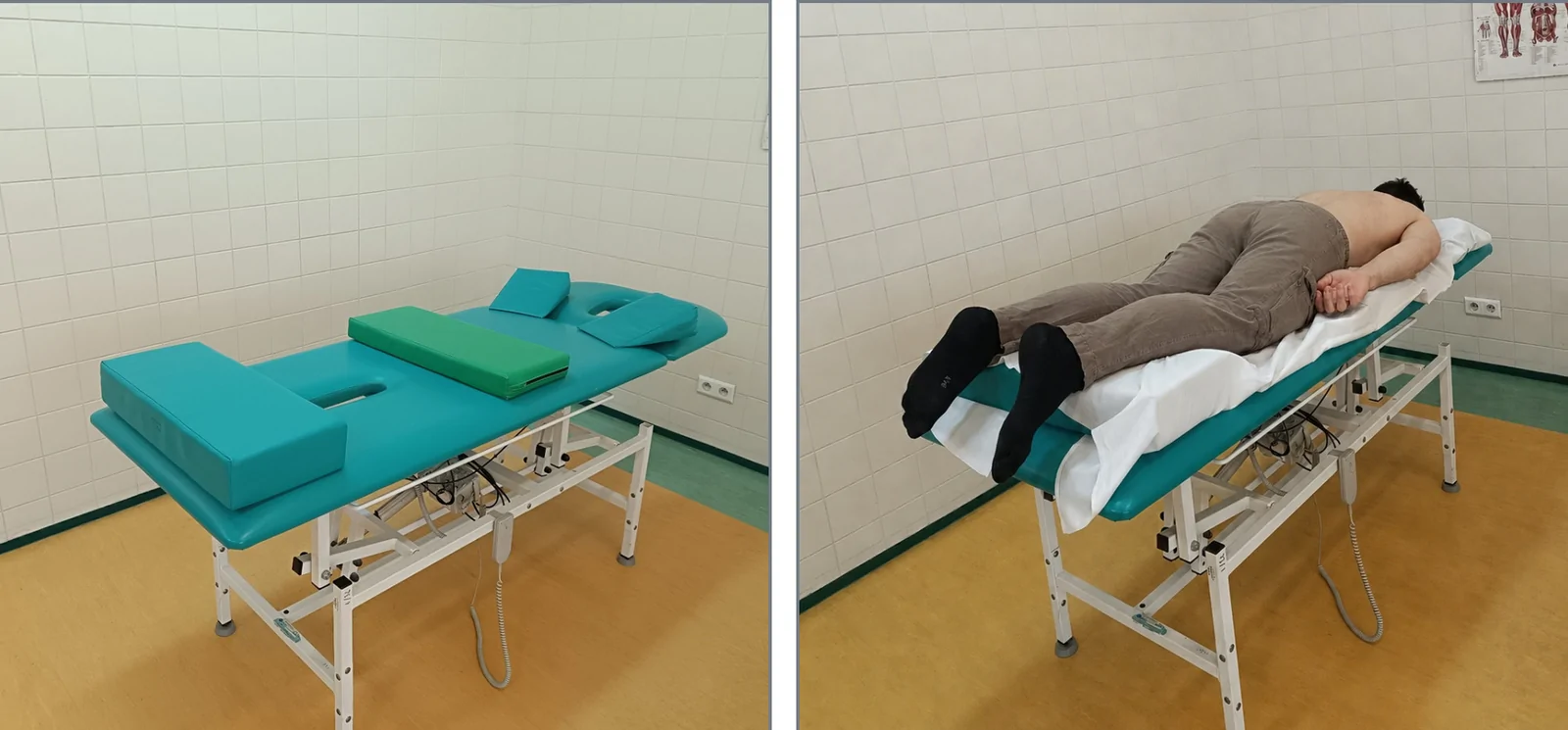
Representative methodological photograph of the recovery setup. The left panel shows the standardized massage-table configuration with positioning wedges before participant placement, and the right panel shows the prone participant position used during the recovery phase. The photograph illustrates body position and support arrangement and was not used for physiological outcome analysis.

As shown in **Figure 3**, positioning was standardized with wedges supporting the ankle/lower-leg region, abdominal region, and shoulder girdle. The upper back was exposed only to the extent required for the intervention, while the remaining body regions were covered or clothed to maintain comfort and reduce unnecessary movement. The arms were positioned comfortably alongside the body, and participants were instructed to remain relaxed and avoid voluntary movement during the recovery period.

The massage area included the posterior trunk, with manual techniques applied bilaterally along the paraspinal region and over the latissimus dorsi, trapezius, and erector spinae muscle regions. Direct pressure over the spinous processes, bony prominences, and painful areas was avoided. The same sequence was used for all participants: preparatory effleurage, tissue displacement, rolling, friction, kneading, and final calming effleurage. Each technique was repeated seven times within the standardized sequence, resulting in a total intervention duration of 15 min.

Massage pressure was maintained at a comfortable, non-painful level and was not intended to provoke pain, stretching discomfort, or strong mechanical stimulation. Massage providers were instructed to adjust pressure only in response to participant-reported discomfort; however, no such discomfort was reported, and no pressure adjustments were required. Verbal communication during the recovery phase was limited to necessary safety or comfort checks, in order to minimize additional cognitive or emotional stimulation. No other relaxation instruction, breathing guidance, music, or cognitive task was provided during the recovery period.

Participants in the control group remained in the same prone position and on the same table setup as the experimental group for the full 15-min recovery period but did not receive massage or any other form of manual stimulation. This condition controlled for recovery duration, body position, and passive post-task rest, while preserving the key experimental contrast related to the presence or absence of structured somatosensory stimulation. Both recovery conditions were conducted in the same room, between 10:00 AM and 3:00 PM, under comparable environmental conditions and similar ambient temperature of approximately 24 °C.

### 2.6 Statistical analysis

Statistical analyses were conducted using jamovi software, version 2.6, based on the R statistical environment. Descriptive statistics were calculated separately for the experimental and control groups. Continuous variables were summarized as means and standard deviations, while categorical variables were presented as frequencies and percentages.

Before inferential analyses, the distribution of continuous variables was evaluated using the Shapiro–Wilk test. Homogeneity of variance between groups was assessed using Levene’s test. Baseline demographic, anthropometric, sleep-related, fatigue-related, stress, and pain/discomfort variables were compared between the experimental and control groups to evaluate baseline comparability. Due to deviations from normality observed for several baseline variables, these comparisons were performed using the Mann–Whitney U test. Sex distribution was compared using the chi-square test. These baseline analyses are reported descriptively in the main manuscript table, with corresponding p values.

Subjective workload induced by the cognitive task was assessed using the six NASA-TLX subscales: mental demand, physical demand, temporal demand, performance, effort, and frustration. Between-group comparisons of NASA-TLX scores were performed using the Mann–Whitney U test. These analyses were used to determine whether the experimental and control groups differed in perceived task-related workload after the cognitive-load induction procedure.

Separate mixed-design repeated-measures ANOVAs were conducted on raw phase mean values for heart rate and mean skin conductance as the primary omnibus analyses. In each model, phase was entered as the within-subject factor and group as the between-subject factor. The main effects of phase and group, together with the phase × group interaction, were examined to determine whether physiological activity changed across the experimental protocol and whether the temporal trajectories differed between the experimental and control groups.

For mixed repeated-measures analyses, results were reported using F values, degrees of freedom, p values, and partial eta squared (ηp²) as the measure of effect size. When the assumption of sphericity was violated, Greenhouse–Geisser correction was applied and corrected degrees of freedom were reported. When a significant phase effect or phase × group interaction was observed, planned follow-up comparisons were used to clarify the direction and temporal location of the effect, with particular attention to baseline versus cognitive task and baseline versus recovery intervals. Pairwise follow-up comparisons for the phase × group interaction were adjusted using the Bonferroni correction, and Bonferroni-adjusted p values are reported for key between-group recovery comparisons. As the study had an exploratory design and several variables deviated from normality, non-parametric analyses were also conducted as supplementary robustness analyses. These included Friedman tests for within-participant phase effects, Durbin–Conover pairwise comparisons for repeated-measures contrasts, and Mann–Whitney U tests for between-group comparisons. Non-parametric results, including test statistics, p values, and corresponding effect-size estimates where applicable, are provided in the **Supplementary Material**.

All statistical tests were two-tailed, and the threshold for statistical significance was set at p <.05. Exact p values were reported where possible. Given the exploratory character of the study, findings were interpreted with attention not only to statistical significance, but also to effect direction, magnitude, and temporal consistency across recovery intervals.

## 3 Results

The baseline characteristics of the experimental and control groups are presented in **Table 1**. No statistically significant between-group differences were observed for sex distribution, age, body height, body mass, BMI, sleep duration, sleep quality, physical fatigue, sleepiness, mental fatigue, stress, or pain/discomfort. Descriptively, both groups showed comparable sleep duration and similar levels of fatigue, stress, and discomfort before the experimental procedure. These analyses did not reveal statistically detectable baseline differences in the measured demographic, anthropometric, sleep-related, fatigue-related, stress-related, or discomfort-related variables.

NASA-TLX and Determination Test performance outcomes are presented in **Table 2**. No statistically significant between-group differences were observed for the NASA-TLX subscales: mental demand, physical demand, temporal demand, performance, effort, or frustration. For DT performance data, no statistically significant between-group differences were observed for median reaction time, correct responses, incorrect responses, omitted responses, or accuracy.

**Table 2:** Between-group comparisons of NASA-TLX workload ratings and determination test performance.

Variable	Experimental group	Control group	Test statistic	p
NASA-TLX mental demand	66.46 ± 15.98	73.54 ± 12.72	U = 218.0	.148
NASA-TLX physical demand	35.42 ± 21.11	46.67 ± 22.59	U = 211.5	.116
NASA-TLX temporal demand	77.29 ± 12.85	78.13 ± 16.93	U = 252.5	.467
NASA-TLX performance	48.54 ± 15.07	53.96 ± 19.78	U = 238.5	.309
NASA-TLX effort	70.21 ± 15.50	72.29 ± 12.85	U = 278.5	.852
NASA-TLX frustration	56.25 ± 25.29	65.00 ± 24.93	U = 228.5	.223
DT median RT	0.729 ± 0.052	0.740 ± 0.057	t(46) = 0.687	.496
DT correct responses	529.38 ± 45.09	514.71 ± 45.18	t(46) = −1.126	.266
DT incorrect responses	21.67 ± 10.26	23.71 ± 16.74	t(46) = 0.509	.613
DT omitted responses	31.63 ± 13.27	29.42 ± 9.48	t(46) = −0.664	.510
DT accuracy	0.908 ± 0.032	0.908 ± 0.030	t(46) = −0.040	.968

Values are presented as mean ± standard deviation. NASA-TLX subscale ratings were compared using Mann–Whitney U tests, whereas DT performance variables were compared using independent-samples Student’s t-tests. Reaction time is expressed in seconds, and accuracy is expressed as the proportion of correct responses. NASA-TLX, NASA Task Load Index; DT, Determination Test; RT, reaction time.

These analyses did not reveal statistically significant between-group differences in the measured task-performance indices during the cognitive-load induction procedure. Corresponding non-parametric sensitivity analyses for the DT indices yielded the same interpretation and did not indicate statistically significant between-group differences.

**Figure 4:**
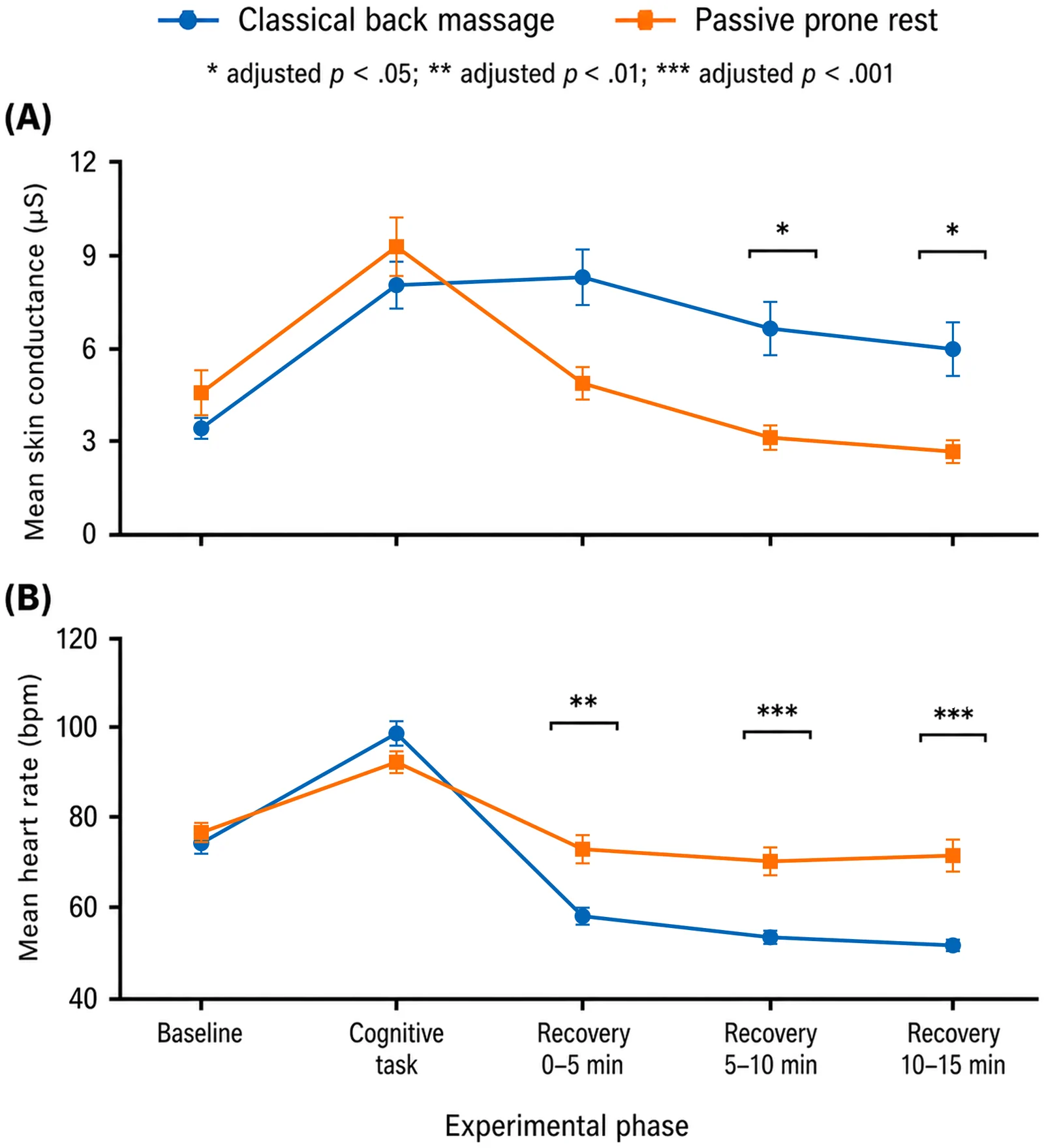
Physiological responses across experimental phases. **(A)** Presents mean skin conductance, and **(B)** presents mean heart rate across baseline, the cognitive task, and three recovery windows. Error bars represent standard errors of the mean (SEM). Asterisks indicate Bonferroni-adjusted between-condition comparisons. µS, microsiemens; bpm, beats per minute.

Mean skin conductance values and baseline-adjusted changes across experimental phases are presented in **Table 3**, **Figure 4A**. Both groups showed increased skin conductance during the cognitive task relative to baseline. During recovery, however, the groups followed different skin-conductance trajectories. In the experimental group, skin conductance remained above baseline throughout all three recovery windows, although it gradually decreased over time. In the control group, skin conductance decreased progressively, approaching baseline during the first recovery window and falling below baseline during the two later windows.

**Table 3:** Skin conductance values and baseline-adjusted changes across experimental phases.

Phase	Experimental group	Experimental Δ vs baseline	Control group	Control Δ vs baseline
Baseline	3.42 ± 1.64	—	4.56 ± 3.55	—
Cognitive task	8.02 ± 3.70	4.61	9.26 ± 4.61	4.70
Recovery 0–5 min	8.28 ± 4.38	4.86	4.86 ± 2.56	0.30
Recovery 5–10 min	6.63 ± 4.20	3.21	3.12 ± 1.92	−1.44
Recovery 10–15 min	5.96 ± 4.23	2.55	2.67 ± 1.79	−1.90

Phase values are presented as mean ± standard deviation. Baseline-adjusted change values were calculated descriptively as phase value − baseline value and are presented as mean differences. Positive values indicate higher mean skin conductance than baseline, whereas negative values indicate lower mean skin conductance than baseline. The primary inferential analyses were performed on raw phase mean values.

Heart rate values and baseline-adjusted changes across experimental phases are presented in **Table 4**, **Figure 4B**. HR increased during the cognitive task in both groups and subsequently decreased during recovery. This reduction was substantially greater in the experimental group, in which HR remained lower than in the control group across all three recovery windows. Baseline-adjusted changes similarly indicated a greater reduction relative to baseline in the experimental group than in the control group.

**Table 4:** Mean PPG-derived heart rate and baseline-adjusted changes across phases in the experimental group (classical back massage) and control group (passive prone rest).

Phase	Experimental group	Experimental Δ vs baseline	Control group	Control Δ vs baseline
Baseline	74.60 ± 11.50	—	76.96 ± 10.45	—
Cognitive task	98.96 ± 13.26	24.37	92.57 ± 11.87	15.61
Recovery 0–5 min	58.38 ± 9.25	−16.21	73.25 ± 15.36	−3.71
Recovery 5–10 min	53.69 ± 7.15	−20.90	70.56 ± 15.17	−6.40
Recovery 10–15 min	51.90 ± 6.23	−22.70	71.84 ± 17.37	−5.12

Phase values are presented as mean ± standard deviation. Baseline-adjusted change values were calculated descriptively as phase value − baseline value and are presented as mean differences. Positive values indicate higher HR than baseline, whereas negative values indicate lower HR than baseline. The primary inferential analyses were performed on raw phase mean values.

The mixed repeated-measures ANOVA results are presented in **Table 5**. For skin conductance, a significant main effect of phase was observed, F(2.24, 103.13) = 67.10, p <.001, ηp² = .593. The phase × group interaction was also significant, F(2.24, 103.13) = 28.50, p <.001, ηp² = .383, indicating that the skin-conductance trajectory differed between the experimental and control groups. The main effect of group was not statistically significant, F(1, 46) = 3.04, p = .088, ηp² = .062.

**Table 5:** Mixed-design repeated-measures ANOVA of phase and post-task condition effects on mean skin conductance and heart rate.

Outcome	Effect	F(df1, df2)	p	ηp²
Mean skin conductance	Phase	67.10 (2.24, 103.13)	<.001	.593
Mean skin conductance	Phase × post-task condition	28.50 (2.24, 103.13)	<.001	.383
Mean skin conductance	Post-task condition	3.04 (1, 46)	.088	.062
HR	Phase	216.60 (2.62, 120.67)	<.001	.825
HR	Phase × post-task condition	32.80 (2.62, 120.67)	<.001	.417
HR	Post-task condition	9.57 (1, 46)	.003	.172

Phase included the resting baseline, cognitive task, and three consecutive post-task windows (0–5, 5–10, and 10–15 min). Post-task condition comprised classical back massage and passive prone rest. Greenhouse–Geisser-corrected degrees of freedom are reported for the main effect of phase and the phase × post-task condition interaction.

For HR, a significant main effect of phase was observed, F(2.62, 120.67) = 216.60, p <.001, ηp² = .825. The phase × group interaction was also significant, F(2.62, 120.67) = 32.80, p <.001, ηp² = .417, indicating that HR changed differently across phases in the two groups. The main effect of group was significant, F(1, 46) = 9.57, p = .003, ηp² = .172. Together, these findings indicate that both physiological outcomes changed across the experimental protocol, while their temporal patterns differed between the massage and passive rest conditions.

Key between-group *post-hoc* comparisons during recovery are presented in **Table 6**. For mean skin conductance, the experimental group showed higher values than the control group across all recovery windows, although the recovery 0–5 min comparison did not remain statistically significant after Bonferroni correction. Significant between-group differences were observed during recovery 5–10 min and recovery 10–15 min. This pattern indicates sustained mean skin conductance during the later post-task windows while massage and tactile stimulation were ongoing. The phase-averaged analysis does not determine whether this pattern reflected a tonic shift, repeated phasic responses, or a combination of both.

**Table 6:** Bonferroni-adjusted *post hoc* comparisons between the classical back massage and passive prone rest conditions for mean skin conductance and heart rate across the three post-task windows.

Outcome	Phase	Mean difference	SE	df	t	Adjusted p
Mean skin conductance	Recovery 0–5 min	3.41	1.04	46	3.30	.085
Mean skin conductance	Recovery 5–10 min	3.51	0.94	46	3.72	.024
Mean skin conductance	Recovery 10–15 min	3.30	0.94	46	3.52	.044
HR	Recovery 0–5 min	−14.87	3.66	46	−4.06	.008
HR	Recovery 5–10 min	−16.87	3.42	46	−4.93	<.001
HR	Recovery 10–15 min	−19.94	3.77	46	−5.30	<.001

Mean differences were calculated as the classical back massage condition minus the passive prone rest condition. Positive values indicate higher values during massage, whereas negative values indicate lower values during massage. Adjusted p values were obtained using the Bonferroni correction.

For HR, the experimental group showed significantly lower values than the control group across all recovery windows: 0–5 min, 5–10 min, and 10–15 min. In the context of this exploratory quasi-randomized design, these *post-hoc* results were consistent with a divergent physiological recovery pattern: lower phase-averaged HR but higher mean skin conductance in the massage condition relative to passive rest.

The primary Bonferroni-adjusted *post hoc* comparisons (**Table 6**) showed higher mean skin conductance in the experimental group across all three recovery windows, with experimental-minus-control differences of 3.41 µS at 0–5 min, 3.51 µS at 5–10 min, and 3.30 µS at 10–15 min. The difference did not reach statistical significance during the 0–5 min window (adjusted p = .085), but skin conductance was significantly higher in the experimental group during the 5–10 min (adjusted p = .024) and 10–15 min windows (adjusted p = .044). Mean HR was lower in the experimental group throughout recovery, with experimental-minus-control differences of −14.87 bpm at 0–5 min, −16.87 bpm at 5–10 min, and −19.94 bpm at 10–15 min. All three HR comparisons were statistically significant (adjusted p = .008, p <.001, and p <.001, respectively). Supplementary non-parametric analyses showed no significant between-group differences in either marker at baseline or during the cognitive task and supported the same overall recovery pattern. Full results are presented in **Supplementary Tables 1**–**5**.

## 4 Discussion

The present study examined cardiovascular and EDA after cognitive load under two post-task conditions: classical massage and passive rest. The main finding was a marker-specific, condition-associated physiological pattern: heart rate was lower during the massage condition, whereas skin conductance remained elevated relative to baseline and was higher than during passive rest in the 5–10 min and 10–15 min windows. These findings describe physiological differences observed under ongoing massage and passive rest but do not isolate a massage-specific effect or demonstrate uniform autonomic downregulation.

Previous studies have reported reductions in HR and blood pressure following massage, together with changes in HRV-derived indices (Moyer et al., 2004; Diego and Field, 2009; Field et al., 2010; Meier et al., 2020). In the present study, participants in the massage condition showed lower phase-averaged HR than those undergoing passive rest. This finding should remain descriptive, as HR alone cannot distinguish increased parasympathetic activity from sympathetic withdrawal or other influences on cardiac rate. The electrodermal results further preclude a uniform interpretation: mean skin conductance decreased toward or below baseline during passive rest but remained elevated during the massage condition. It should be noted, however, that baseline was recorded in a seated position, whereas recovery was performed in a prone position in both groups. Therefore, baseline-to-recovery HR decreases cannot be attributed solely to the massage condition. The most relevant interpretation concerns the between-group differences during recovery, as both groups underwent the same postural transition and remained prone for the same duration. Nevertheless, this between-condition comparison still represents an active manual and interpersonal condition versus no-touch passive rest and therefore does not isolate the specific contribution of classical massage techniques.

This electrodermal pattern partly contrasts with previous studies reporting reductions in skin conductance or sympathetic skin-response indices after massage or massage-like interventions (Zullino et al., 2005; Lee et al., 2011; Kerautret et al., 2024). The discrepancy may reflect differences in intervention context, timing, stimulation type, and outcome assessment. In the present study, massage was delivered immediately after a cognitively demanding task and skin conductance was assessed during recovery from that task; several earlier studies examined massage as a general relaxation or well-being intervention without an immediately preceding cognitive-load induction (Zullino et al., 2005; Kerautret et al., 2024).

One plausible explanation concerns the sensory properties of massage. Skin conductance primarily reflects sympathetic sudomotor activity and is also sensitive to orienting, emotional salience, stimulus processing, and sensory input (Critchley, 2002; Dawson et al., 2007; Boucsein, 2012). It should therefore not be interpreted as a general measure of sympathetic activation or stress. Sustained skin conductance during massage may reflect responsiveness to ongoing somatosensory input, although the present phase-averaged analysis cannot distinguish tonic changes from repeated phasic responses. Importantly, EDA in the present study was recorded throughout ongoing massage rather than during a stimulation-free post-intervention period. Experimental evidence indicates that stroking can produce greater skin conductance responses and levels than tapping, while light-touch stimulation of C-tactile afferents can itself elicit a sympathetic skin response (Olausson et al., 2008; Etzi et al., 2018). The higher phase-averaged skin conductance observed in the massage condition may therefore have reflected repeated responses to successive strokes, changes in pressure, or other sensory events rather than delayed autonomic recovery. As recording was not continued after massage had ceased, the study cannot determine whether EDA subsequently returned toward baseline. Accordingly, elevated skin conductance should be interpreted as a concurrent electrodermal response under ongoing tactile stimulation rather than evidence of impaired or delayed recovery. Recent affective-touch evidence supports this interpretation. Slow stroking and rhythmic touching can decrease HR and increase parasympathetic-related indices while also increasing skin conductance level (Köteles et al., 2024), and touch responses depend on stimulus properties and affective somatosensory processing (Ackerley et al., 2014; McGlone et al., 2014). Therefore, tactile input may be accompanied by divergent patterns across HR and skin conductance rather than a uniform decrease in both measures.

The findings also highlight the need for multimodal physiological interpretation in cognitive-load and recovery research. Reviews emphasize that single markers cannot serve as universal indicators of cognitive load, stress, or recovery across tasks and contexts (Charles and Nixon, 2019; Tao et al., 2019). In the present study, considering heart rate alone would emphasize the lower cardiovascular values observed during the massage condition, whereas considering skin conductance alone would emphasize sustained EDA. The combined pattern is more informative but should remain descriptive and condition-specific.

The findings therefore support a cautious, condition-based interpretation. Review-level evidence shows heterogeneous effects across massage techniques, populations, pressures, durations, comparator conditions, and outcome measures (Moyer et al., 2011; Nelson, 2015; Field, 2016). In the present study, lower heart rate occurred alongside sustained mean skin conductance during ongoing massage relative to passive rest. If replicated using randomized allocation and an active tactile comparator, this pattern may have practical relevance for the post-task period. The current design, however, cannot determine whether the observed differences reflect classical massage techniques, non-specific tactile stimulation, therapist interaction, expectation, or other contextual factors.

## 5 Limitations

Several limitations should be considered when interpreting the present findings.

Most importantly, the control condition consisted of passive prone rest without manual stimulation. The study therefore compared a combined manual and interpersonal condition with no-touch passive rest and did not isolate the specific contribution of classical massage techniques from somatosensory stimulation, therapist interaction, expectation, attention directed toward the body, or the broader context of receiving a manual intervention. Accordingly, the observed between-condition differences cannot be attributed specifically to classical massage. Future studies should include active comparators such as light touch, sham massage, or standardized non-therapeutic tactile stimulation.

Although the final physiological analytical sample exceeded the minimum sample size estimated in the *a priori* power analysis, the study retained an exploratory character. The sample consisted of healthy young adults, which limits generalization to older adults, clinical populations, individuals with chronic stress, or professional performance groups exposed to repeated cognitive load. Two experimental-group recordings were excluded due to extensive physiological artefacts, which additionally reduces the effective sample size for the primary analyses and should be considered when interpreting the robustness of between-group effects.

The study used a single-session intervention. The results describe acute cardiovascular and electrodermal responses to a single classical massage session after cognitive load. They do not allow conclusions regarding repeated massage exposure, long-term adaptation, or sustained recovery effects across days or weeks.

EDA is highly sensitive to multiple influences, including skin properties, thermoregulation, electrode contact, hydration, emotional salience, novelty, attention, and sensory input. Although accelerometer and gyroscope channels were retained to support movement artefact control, inertial monitoring cannot eliminate all possible sources of physiological or technical artefact. Therefore, the elevated skin conductance observed during massage should be interpreted cautiously as a skin-conductance response occurring during a manual intervention, rather than as a direct marker of persistent stress.

The use of a predetermined alternating allocation sequence represents an additional limitation. As the subsequent assignment was predictable, the procedure did not provide allocation concealment and may have introduced selection bias, thereby reducing internal validity. Although no statistically significant between-condition differences were identified in the measured baseline characteristics, this does not exclude imbalances in unmeasured factors or residual confounding. Consequently, the findings do not support causal inference. Future confirmatory studies should use computer-generated randomization and concealed allocation.

A further limitation concerns possible inter-provider variability in the massage intervention. Although the intervention followed a standardized protocol, provider-specific differences could not be fully eliminated. This is particularly relevant for applied pressure, which was standardized pragmatically as comfortable and non-painful, but was not instrumentally quantified or formally calibrated. Subtle differences in pressure magnitude, rhythm, movement velocity, hand contact, and manual handling may have contributed to between-participant variability, especially in skin conductance recorded during massage. Future studies should consider objective pressure monitoring, formal pressure calibration, recording provider identity for each participant, or modelling provider as an additional factor in larger samples.

Finally, the physiological analysis focused on PPG-derived phase-averaged HR and phase-averaged mean skin conductance. Moreover, the phase-averaged mean skin-conductance outcome did not allow a sustained tonic shift to be distinguished from repeated phasic responses to ongoing tactile stimulation. Mean HR cannot distinguish sympathetic withdrawal from parasympathetic activation and therefore does not provide direct evidence of cardiac autonomic modulation. Although PPG-derived inter-beat intervals were recorded automatically, they were not subjected to the beat-level validation and artefact-correction procedures required for reliable PRV analysis, particularly during ongoing manual stimulation. Respiration was not recorded, which further limited the interpretation of frequency-domain PRV measures. The findings should therefore be interpreted as global cardiovascular and electrodermal phase responses rather than evidence of specific autonomic mechanisms. Future studies should incorporate electrocardiography-derived HRV or validated PPG-derived PRV indices, such as the root mean square of successive differences, the standard deviation of normal-to-normal intervals, and frequency-domain measures, together with tonic–phasic EDA decomposition.

## 6 Conclusion

The present exploratory pilot study identified condition-associated differences in physiological responses after cognitive load. Heart rate was lower during the massage condition than during passive rest, whereas skin conductance remained elevated during massage and was higher than during passive rest in the later post-task windows. These findings indicate a marker-specific physiological pattern under two different post-task conditions rather than uniform autonomic deactivation or massage-specific improvement in recovery. Accordingly, heart rate and mean skin conductance should not be interpreted as interchangeable indicators of physiological state. As EDA was summarized using phase-averaged mean skin conductance without tonic–phasic decomposition, the elevated values cannot be attributed specifically to either sustained tonic activity or repeated phasic responses to ongoing tactile stimulation.

Within the present quasi-randomized design, the massage condition should be conceptualized as an active manual condition involving ongoing somatosensory and interpersonal stimulation rather than as a passive relaxation state. The findings should be treated as preliminary and hypothesis-generating. Future randomized studies incorporating active tactile or sham control conditions are needed to determine whether the observed pattern is attributable to massage-specific techniques, reproducible, and practically relevant after cognitive demand.

## Data Availability

The original contributions presented in the study are included in the article/**Supplementary Material**. Further inquiries can be directed to the corresponding author.

## Supplementary material

Supplementary Figure 1 Representative synchronized physiological recordings from an original study session. **(A)** shows a 30-s segment recorded during massage recovery, and **(B)** shows a 30-s movement-disturbed segment from the task-to-recovery transition.
