# Supplementary material for: Classical back massage is associated with lower heart rate and sustained mean skin conductance during recovery after cognitive load: an exploratory controlled pilot study
Source: Front Physiol. 2026 Sep 8;17:1917630. doi: 10.3389/fphys.2026.1917630 (PMC13593411; doi:10.3389/fphys.2026.1917630)
Supplement: Supplementary file 1 [file SupplementaryFile1.pdf]

Senacka Komisja ds. Etyki Badań Naukowych
przy Akademii Wychowania Fizycznego
im. Polskich Olimpijczyków we Wrocławiu

Uchwała nr 46/2025
w sprawie opinii o projekcie eksperymentu poznawczego

Na podstawie Uchwał Senatu Akademii Wychowania Fizycznego im. Polskich Olimpijczyków we Wrocławiu: nr 74/2002 z dnia 20.12.2002 r. w sprawie powołania Senackiej Komisji ds. Etyki Badań Naukowych i nr 26/2024 z dnia 23.05.2024 r. w sprawie przyjęcia Regulaminu Senackiej Komisji ds. Etyki Badań Naukowych oraz w oparciu o: art. 27 ustawy z dnia 6.06.1997 r. kodeks karny (Dz.U. z 1997 r., poz.553 z późniejszymi zmianami), zasady zawarte w „Kodeksie Etyki Pracownika Naukowego (Wydanie III)" z dn. 25.06.2020 r. i w „Kodeksie Etyki Pracownika Naukowego Akademii Wychowania Fizycznego im. Polskich Olimpijczyków we Wrocławiu" z dn. 29.02.2024 r.

Przewodniczący Senackiej Komisji ds. Etyki Badań Naukowych
przy Akademii Wychowania Fizycznego im. Polskich Olimpijczyków we Wrocławiu
po zapoznaniu się z opinią Członków Komisji na temat wniosku złożonego przez Pana:

Dra Macieja Lachowicza

pt. „Zastosowanie masażu jako metody redukcji przeładowania poznawczego: badania pilotażowe"

podjął decyzję o pozytywnym zaopiniowaniu tego wniosku, nie wnosząc żadnych zastrzeżeń.

Wydana opinia dotyczy tylko rozpatrywanego wniosku z uwzględnieniem przedstawionego projektu. Każda zmiana i modyfikacja wymaga uzyskania odrębnej opinii. Wnioskodawca zobowiązany jest do informowania o ciężkich lub niespodziewanych zdarzeniach, niepożądanych i nieprzewidzianych okolicznościach, o zakończeniu badania, o jego wynikach i innych istotnych decyzjach ewentualnych innych komisji etycznych (bioetycznych).

Od powyższej uchwały podmiot zamierzający przeprowadzić eksperyment, kierownik jednostki organizacyjnej, w którym eksperyment poznawczy ma być przeprowadzony, oraz komisja etyczna (bioetyczna) właściwa dla ośrodka, który ma ewentualnie uczestniczyć w wieloośrodkowym eksperymencie, mogą wnieść odwołanie do Zespołu Opiniodawczo-Doradczego do Spraw Etyki w Nauce Ministerstwa Nauki i Informatyzacji, za pośrednictwem Senackiej Komisji ds. Etyki Badań Naukowych przy Akademii Wychowania Fizycznego im. Polskich Olimpijczyków we Wrocławiu w terminie 14 dni od daty otrzymania niniejszej uchwały.

Przewodniczący Senackiej Komisji
ds. Etyki Badań Naukowych
Pezdek
Dr hab. Krzysztof Pezdek, prof. AWF

Wrocław, dnia 28.01.26 r.
