# Supplementary material for: Classical back massage is associated with lower heart rate and sustained mean skin conductance during recovery after cognitive load: an exploratory controlled pilot study
Source: Front Physiol. 2026 Sep 8;17:1917630. doi: 10.3389/fphys.2026.1917630 (PMC13593411; doi:10.3389/fphys.2026.1917630)
Supplement: Supplementary file 2 [file DataSheet1.docx]

**Supplementary non-parametric analyses**

**Supplementary Table 1. Non-parametric between-group comparisons: Mann-Whitney U tests**

| **Variable** | **U** | **p** | **Rank-biserial correlation** |
| --- | --- | --- | --- |
| GSR baseline | 260.0 | .571 | -0.097 |
| GSR cognitive task | 244.0 | .370 | -0.153 |
| GSR recovery 0-5 min | 156.0 | .007 | 0.458 |
| GSR recovery 5-10 min | 125.0 | < .001 | 0.566 |
| GSR recovery 10-15 min | 125.0 | < .001 | 0.566 |
| HR baseline | 263.0 | .613 | -0.087 |
| HR cognitive task | 205.0 | .089 | 0.288 |
| HR recovery 0-5 min | 111.0 | < .001 | -0.615 |
| HR recovery 5-10 min | 64.0 | < .001 | -0.778 |
| HR recovery 10-15 min | 53.0 | < .001 | -0.816 |

Note. Between-group comparisons were performed with Mann-Whitney U tests. For interpretive clarity, rank-biserial correlations were sign-oriented so that positive values indicate higher values in the experimental group and negative values indicate lower values in the experimental group. Descriptive group values indicate higher GSR in the experimental group during recovery and lower HR in the experimental group during recovery.

**Supplementary Table 2. Non-parametric repeated-measures analyses in the experimental group: Friedman tests**

| **Outcome** | **Friedman chi-square** | **df** | **p** |
| --- | --- | --- | --- |
| GSR | 61.5 | 4 | < .001 |
| HR | 88.8 | 4 | < .001 |

*Note.* Friedman tests were performed across baseline, cognitive task, recovery 0-5 min, recovery 5-10 min, and recovery 10-15 min.

**Supplementary Table 3. Pairwise Durbin-Conover comparisons in the experimental group**

| **Outcome** | **Comparison** | **Statistic** | **p** |
| --- | --- | --- | --- |
| GSR | Baseline - cognitive task | 10.286 | < .001 |
| GSR | Baseline - recovery 0-5 min | 10.882 | < .001 |
| GSR | Baseline - recovery 5-10 min | 6.857 | < .001 |
| GSR | Baseline - recovery 10-15 min | 4.025 | < .001 |
| GSR | Cognitive task - recovery 0-5 min | 0.596 | .552 |
| GSR | Cognitive task - recovery 5-10 min | 3.429 | < .001 |
| GSR | Cognitive task - recovery 10-15 min | 6.261 | < .001 |
| GSR | Recovery 0-5 min - recovery 5-10 min | 4.025 | < .001 |
| GSR | Recovery 0-5 min - recovery 10-15 min | 6.857 | < .001 |
| GSR | Recovery 5-10 min - recovery 10-15 min | 2.832 | .006 |
| HR | Baseline - cognitive task | 7.85 | < .001 |
| HR | Baseline - recovery 0-5 min | 8.83 | < .001 |
| HR | Baseline - recovery 5-10 min | 17.33 | < .001 |
| HR | Baseline - recovery 10-15 min | 20.93 | < .001 |
| HR | Cognitive task - recovery 0-5 min | 16.68 | < .001 |
| HR | Cognitive task - recovery 5-10 min | 25.18 | < .001 |
| HR | Cognitive task - recovery 10-15 min | 28.78 | < .001 |
| HR | Recovery 0-5 min - recovery 5-10 min | 8.50 | < .001 |
| HR | Recovery 0-5 min - recovery 10-15 min | 12.10 | < .001 |
| HR | Recovery 5-10 min - recovery 10-15 min | 3.60 | < .001 |

**Supplementary Table 4. Non-parametric repeated-measures analyses in the control group: Friedman tests**

| **Outcome** | **Friedman chi-square** | **df** | **p** |
| --- | --- | --- | --- |
| GSR | 78.7 | 4 | < .001 |
| HR | 60.3 | 4 | < .001 |

*Note.* Friedman tests were performed across baseline, cognitive task, recovery 0-5 min, recovery 5-10 min, and recovery 10-15 min.

**Supplementary Table 5. Pairwise Durbin-Conover comparisons in the control group**

| **Outcome** | **Comparison** | **Statistic** | **p** |
| --- | --- | --- | --- |
| GSR | Baseline - cognitive task | 10.75 | < .001 |
| GSR | Baseline - recovery 0-5 min | 4.21 | < .001 |
| GSR | Baseline - recovery 5-10 min | 3.79 | < .001 |
| GSR | Baseline - recovery 10-15 min | 8.01 | < .001 |
| GSR | Cognitive task - recovery 0-5 min | 6.53 | < .001 |
| GSR | Cognitive task - recovery 5-10 min | 14.54 | < .001 |
| GSR | Cognitive task - recovery 10-15 min | 18.75 | < .001 |
| GSR | Recovery 0-5 min - recovery 5-10 min | 8.01 | < .001 |
| GSR | Recovery 0-5 min - recovery 10-15 min | 12.22 | < .001 |
| GSR | Recovery 5-10 min - recovery 10-15 min | 4.21 | < .001 |
| HR | Baseline - cognitive task | 4.836 | < .001 |
| HR | Baseline - recovery 0-5 min | 3.664 | < .001 |
| HR | Baseline - recovery 5-10 min | 6.301 | < .001 |
| HR | Baseline - recovery 10-15 min | 4.396 | < .001 |
| HR | Cognitive task - recovery 0-5 min | 8.500 | < .001 |
| HR | Cognitive task - recovery 5-10 min | 11.137 | < .001 |
| HR | Cognitive task - recovery 10-15 min | 9.232 | < .001 |
| HR | Recovery 0-5 min - recovery 5-10 min | 2.638 | .010 |
| HR | Recovery 0-5 min - recovery 10-15 min | 0.733 | .466 |
| HR | Recovery 5-10 min - recovery 10-15 min | 1.905 | .060 |
